# Cyclodextrinase from *Thermococcus* sp expressed in *Bacillus subtilis* and its application in the preparation of maltoheptaose

**DOI:** 10.1186/s12934-020-01416-y

**Published:** 2020-08-01

**Authors:** Lei Wang, Quan Wu, Kang Zhang, Sheng Chen, Zhengfei Yan, Jing Wu

**Affiliations:** 1 State Key Laboratory of Food Science and Technology, Jiangnan University, 1800 Lihu Avenue, Wuxi, 214122 China; 2 School of Biotechnology and Key Laboratory of Industrial Biotechnology Ministry of Education, Jiangnan University, 1800 Lihu Avenue, Wuxi, 214122 China; 3 International Joint Laboratory on Food Safety, Jiangnan University, 1800 Lihu Avenue, Wuxi, 214122 China

**Keywords:** *Thermococcus* sp B1001, Cyclodextrinase, Malto-oligosaccharides, Maltoheptaose

## Abstract

**Background:**

Maltoheptaose as malto-oligosaccharides with specific degree of polymerization, has wide applications in food, medicine and cosmetics industries. Currently, cyclodextrinase have been applied as prepared enzyme to prepare maltoheptaose. However, the yield and proportion of maltoheptaose was lower, which is due to limited substrate and product specificity of cyclodextrinase (CDase). To achieve higher maltoheptaose yield, cyclodextrinase with high substrate and product specificity should be obtained.

**Results:**

In this study, cyclodextrinase derived from *Thermococcus* sp B1001 (TsCDase) was successfully expressed and characterized in *Bacillus subtilis* for the first time. The specific activity of TsCDase was 637.95 U/mg under optimal conditions of 90 °C and pH 5.5, which exhibited high substrate specificity for cyclodextrins (CDs). When the concentration of β-CD was 8%, the yield of maltoheptaose achieved by TsCDase was 82.33% across all reaction products, which exceeded the yields of maltoheptaose in other recent reports. Among malto-oligosaccharides generated as reaction products, maltoheptaose was present in the highest proportion, about 94.55%.

**Conclusions:**

This study provides high substrate and product specificity of TsCDase. TsCDase is able to prepare higher yield of maltoheptaose through conversion of β-CD in the food industry.

## Background

Malto-oligosaccharides are composed of 2–10 glucose units connected by α-1,4 linkages. They are considered to be important functional oligosaccharides that are beneficial for digestion and absorption in humans. Due to their good adaptability, malto-oligosaccharides have been widely applied as food additives in the food industry to improve the properties of food products, such as sweetness, hygroscopicity, stability, viscosity, and gelation [1, 2]. Malto-oligosaccharides are usually obtained from starch as substrate by malto-oligosaccharide-forming amylases [3]. However, adopting this route may yield malto-oligosaccharides with different polymerization degrees of oligosaccharides, resulting in the complex mixtures. Currently, it is difficult to prepare malto-oligosaccharides with specific degrees of polymerization using malto-oligosaccharide-forming amylases, because of the lack of substrate and product specificity.

At present, malto-oligosaccharides with a specific degree of polymerization, including maltotriose, maltotetraose and maltoheptaose, have a specific application, such as fast energy supply, anti-starch aging, and lowering osmotic pressure. Compared with that of maltotriose and maltotetraose, maltoheptaose has a lower osmotic pressure, higher viscosity, better moisturizing effect, and stronger film-forming performance, so that it has been widely applied in the food, medicine, cosmetics, and other fields [4], as well as being a saccharides-based candidate for block copolymers [5]. Thus, an investigation of enzymes with the substrate and product specificity is essential to achieve the mass production of high purity maltoheptaose.

There have so far been various reports of several malto-oligosaccharide-forming amylases that hydrolyze substrates to form maltoheptaose. Among substrates, cyclodextrins (CDs) are the most commonly used including α-CD, β-CD, and γ-CD, which are composed of glucose units linked through α-d-(1,4)-glycosidic bonds. Three types of enzymes have been identified that efficiently hydrolyze CDs: cyclomaltodextrinase (CDase, EC 3.2.1.54), neopullulanase (NPase, EC 3.2.1.135), and maltogenic amylase (MAase, EC 3.2.1.133) [6]. These enzymes also hydrolyze other substrates, such as starch and pullulan, but with slower hydrolytic activity. This is may be due to 130 residues at the N-terminus that might influence that catalytic efficiency of the active domain, and redundant 70 redundant residues at the C-terminus that are absent in the α-amylases. As their most preferred substrates are CDs, these enzymes have been proposed to have a single name, cyclodextrinase (CDases) [6]. At present, whole genome sequence analysis has been confirmed that CDases exist widely in various microorganisms, such as *Bacillus coagulans* [7], *Anoxybacillus flavithermus* [8], and *Bacillus clarkii* [9].

To date, heterologous expression of CDases has been investigated by using *Escherichia coli* only as the host. However, *E. coli* is an unsuitable host for use in the food industry, because of endotoxin production. *Bacillus subtilis* is a generally identified as a safe (GRAS) organism as it does not secrete toxins. *B. subtilis* as an emerging host exhibits no obvious codon preference and is capable of expression products secretion without inclusion bodies.

CDases have been reported to be a very effective enzyme for the production of maltoheptaose. Ji et al. described a novel CDase from *Palaeococcus pacificus* that prepare high purity maltoheptaose. Sequence alignment of CDase showed that *Thermococcus* sp B1001 CDase (TsCDase) have a high degree of similarity with *P. pacificus* CDase (Additional file 1: Figure S1). Previous studies also reported that TsCDase has higher catalytic efficiency for β-CD than other substrates [10, 11]. Thus, TsCDase may be a good candidate for maltoheptaose production using β-CD as substrate. We chose to selecte *Bacillus subtilis* WS9 as the host strain for expression of TsCDase, and recombinant TsCDase was characterized in detail. The ability of TsCDase to produce high purity maltoheptaose using different concentrations of β-CD was also investigated. Compared with other CDases, TsCDase has achieved higher yield and purity maltoheptaose at high concentration of β-CD.

## Materials and methods

### Reagents and chemicals

Maltoheptaose, α-CD, β-CD, γ-CD, soluble starch, and pullulan polysaccharide were purchased from Sigma-Aldrich (Shanghai, China). Taq DNA polymerase, protein and DNA size markers were purchased from Takara (Dalian, China). Unless otherwise noted, other chemicals were obtained from Sinopharm (Shanghai, China).

### Strains and plasmids

The cyclodextrin hydrolase gene (*tscd*) from *Thermofocus* sp B1001 (GenBank accession number: BAB18100.1) was synthesized using pET-24a chemically, and named pET-24a-*tscd*. *E. coli* JM109 was preserved in our laboratory for recombinant plasmid replication. *B. subtilis* WS9 and expression vector pUB110 were obtained from our laboratory for TsCDase expression.

### Construction of expression plasmids

**Fig. 1:**
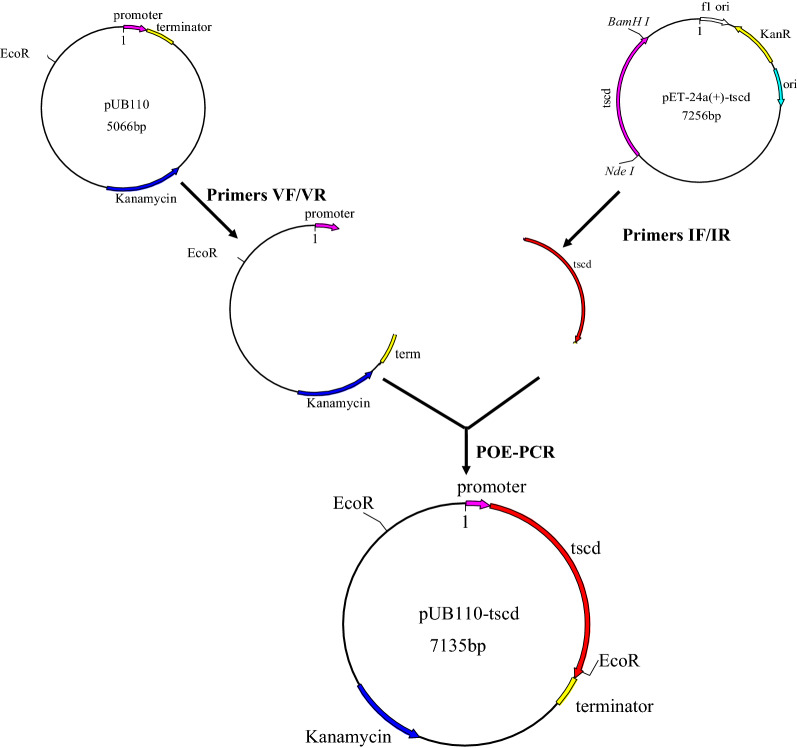
The construction of recombinant plasmid pUB110-*tscd*

Two target fragments were amplified using pET-24a-*tscd* (primers IF/IR) and pUB110 (primers VF/VR) as templates, respectively. Based on these target fragments, the DNA multimers were generated to form recombinant plasmid pUB110-*tscd* (Fig. 1) by prolonged overlap extension PCR (POE-PCR) [12], which was duplicated in *E. coli* JM109. Recombinant plasmid pUB110-*tscd* was verified by sequencing, followed by expression in *B. subtilis* WS9 by the electroporation method [13]. *B. subtilis* WS9 with pUB110 was used applied as a control to determine successful TsCDase expression. All designed primers are listed in Table 1.

**Table 1:** Primers used to amplify the target gene and vector

Primers	Sequences
IF	TAAGAAAATGAGAGGGAGAGGAAACATGTATAAAATTTTTGGCTTTAAAG
IR	AGCTTGGAGGTGTTTTTTTATTACCGCTTTGTTAGCAGCCGGATCTCAGT
VF	ACTGAGATCCGGCTGCTAACAAAGCGGTAATAAAAAAACACCTCCAAGCT
VR	CTTTAAAGCCAAAAATTTTATACATGTTTCCTCTCCCTCTCATTTTCTTA

Homologous arms are underlined

### Production of TsCDase in *B. subtlis* WS9 and its purification

A single colony of recombinant *B. subtlis* WS9 with pUB110-*tscd* was incubated as a seed culture in LB broth containing 100 μg/mL kanamycin, at 37 °C, 200 rpm for 8 h. The seed culture (2% v/v) was added into 50 mL TB broth with 100 μg/mL kanamycin, at 37 °C, 200 rpm for 2 h, and then cultured for another 24 h at 33 °C. To purify TsCDase, whole-cell pellets were obtained by centrifuging at 12,000×*g* and 4 °C for 20 min. They were then re-suspended in 50 mM Tris–HCl (pH 7.5) with 20 mg/mL lysozyme at 37 °C for 30 min, followed by treatment in a high-pressure homogenizer. The supernatant of lysed cells that was obtained by centrifuging at 20,000×*g* and 4 °C for 10 min, was heated at 90 °C for 15 min to precipitate denatured proteins. The pre-purified supernatant containing soluble TsCDase was obtained by centrifuging at 20,000×*g* and 4 °C for 20 min, and was further purified using a nickel-nitrilotriacetic acid (Ni–NTA) column. Bound protein was eluted with a linear gradient of 0–300 mM imidazole in 50 mM Tris–HCl, 300 mM NaCl, at pH 7.5. Purified protein was verified by sodium dodecyl sulfate–polyacrylamide gel electrophoresis (SDS-PAGE) analysis [14], and its concentration was estimated by the Bradford method [15].

### Enzyme activity of TsCDase

The enzyme activity of TsCDase was measured, according to the sum of reducing sugars. In the assay mixture, 1.9 mL of 2% β-CD (w/v) was pre-treated in 50 mM Tris–HCl buffer (pH 7.5) at 90 °C for 10 min, and then mixed with 100 μL TsCDase for 10 min. After stopped by ice water, reducing sugars were estimated by dinitrosalicylic acid (DNS) method [16].

### Effect of pH and temperature on TsCDase activity

To determine optimal pH of TsCDase, relative activity of TsCDase in buffers of various pH values (4.0–8.0) were investigated at 90 °C by method described above. TsCDase was incubated at pH 5.0–9.0 and 4 °C for 24 h, and relative activity was determined to measure pH stability at 90 °C by method described above. The optimal temperature of TsCDase activity was determined at different temperatures (75–100 °C) in 50 mM pH 7.5 Tris–HCl buffer by method described above. The thermostability of TsCDase was determined by incubating TsCDase for 180 min at optimal temperature in 50 mM pH 7.5 Tris–HCl buffer, followed by cooling the solution on ice water, and relative activity was determined at 30 min intervals by method described above.

### Substrate specificity of TsCDase

The substrate specificity of TsCDase was determined against α-CD, β-CD, γ-CD, soluble starch and pullulan polysaccharide. These substrates were dissolved in 50 mM sodium phosphate buffer (pH 6.0) at a final concentration of 1% (w/v), and after the addition of TsCDase (0.3 μg), incubated at 90 °C for 10 min. After stopping the reaction by ice water, and then relative activity of TsCDase was determined by method described above.

### Preparation of maltoheptaose using TsCDase

TsCDase was used to prepare maltoheptaose with β-CD as substrate. The effect of different concentrations of β-CD (2%, 4%, 6%, 8%, 10%, and 12% w/v) on the preparation of maltoheptaose was investigated at 90 °C, 150 rpm, pH 5.5 for 4 h by adding TsCDase (2.8 U) to a final volume of 30 mL. The enzymatic reaction was stopped by adding 250 μL of 0.4 M NaOH, followed by neutralization with 0.4 M HCl. Excess β-CD was removed as a precipitate from the reaction mixture after adding acetonitrile for 2 h at room temperature. The supernatant that collected by centrifuging at 20,000×*g* and 4 °C for 20 min, was characterized by HPLC as described below.

### Identification of the reaction products by HPLC

The supernatant was detected by HPLC using a RID-10A detector, and an APS-2 HYPERSIL column (250 mm × 4.6 mm, Agilent) was used at 30 °C. Acetonitrile/water (80:20, v/v) was applied as the mobile phase with a flow rate of 0.8 mL/min.

### Statistical analysis

All experiments were repeatedly carried out three times, and the results are presented as the means ± standard deviations.

## Results and discussion

### Gene expression in *B. subtilis* WS9 and purification of TsCDase

**Fig. 2:**
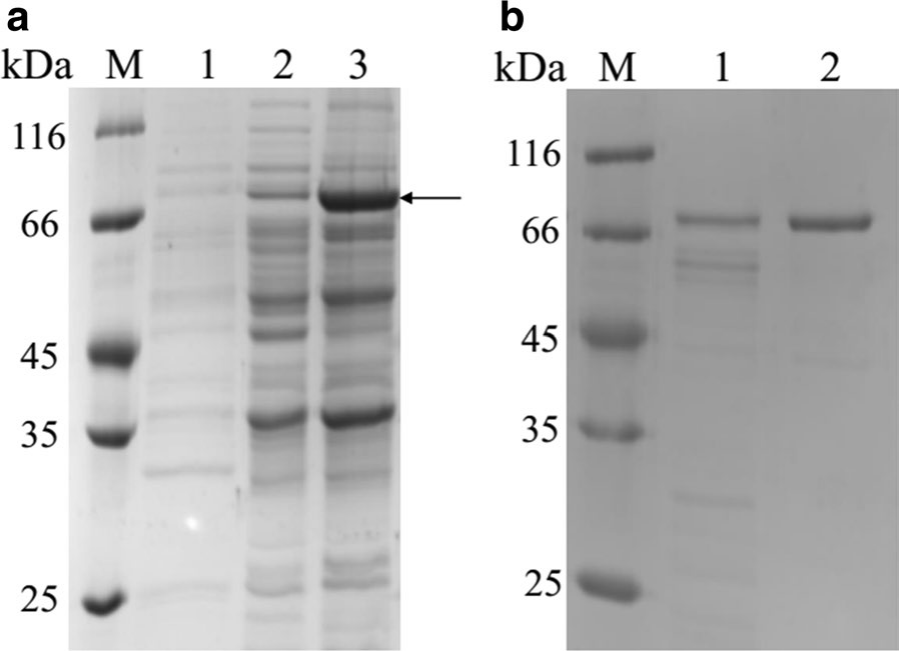
SDS-PAGE analysis of TsCDase **a** and protein purification **b** expressed by *B. subtilis*. **a** M: Marker; 1: Extracellular supernatant; 2: Supernatant of lysed cells; 3: Sediment of lysed cells; **b** M: Marker; 1: Pre-purified enzyme after heat treatment (); 2: Purified TsCDase after Ni–NTA column

TsCDase from *Thermococcus* sp B1001 (approximately 66 kDa) was first successfully expressed in *B. subtilis* WS9, which was widely found in the intracellular compartment of recombinant *B. subtilis* WS9 (Fig. 2). Only partial TsCDase as soluble protein were in the supernatant of lysed cells, whose activity was approximately 5.9 U/mL with specific activity of 45.24 U/mg (Fig. 2a Lane 2). Most TsCDase as inclusion bodies in sediment of lysed cells, but with no activity (Fig. 2a Lane 3). TsCDase in the supernatant of lysed cells was purified with a combined purification protocol by heat treatment and a Ni–NTA column (Fig. 2b). After heat treatment at 90 °C for 15 min, the level of recovery was 91.47% with 76.75 U/mg of protein. This might be because the heat treatment was able to denature and precipitate almost proteins from the host to improve the purity of soluble TsCDase in the soluble fraction, which was consistent with previous study [17]. Meanwhile, high temperature also could promote the formation of proper protein folding of TsCDase, which could enhance its enzyme activity. Without heat treatment, TsCDase was in an intermediate stage of protein folding with slight activity, which was also observed in CDases from *Pyrococcus furiosus* and *P. pacificus* [17, 18]. Subsequently, TsCDase was further purified using a Ni–NTA column with 637.95 U/mg of protein (Table 2). TsCDase activity was greater than that of CDase described by Li et al., as well as its specific activity [19].

**Table 2:** Summary of the steps involved in purification of recombinant TsCDase

Purification step	Total protein (mg)	Total activity (U)	Specific activity (U/mg)	Purification-fold	Yield (%)
Cell lysate	118.85	5376.83	45.24	1	100
Heat treatment	64.08	4918.19	76.75	1.7	91.47
Ni–NTA column	1.31	835.72	637.95	14.1	17

### Effect of pH and temperature on TsCDase activity

**Fig. 3:**
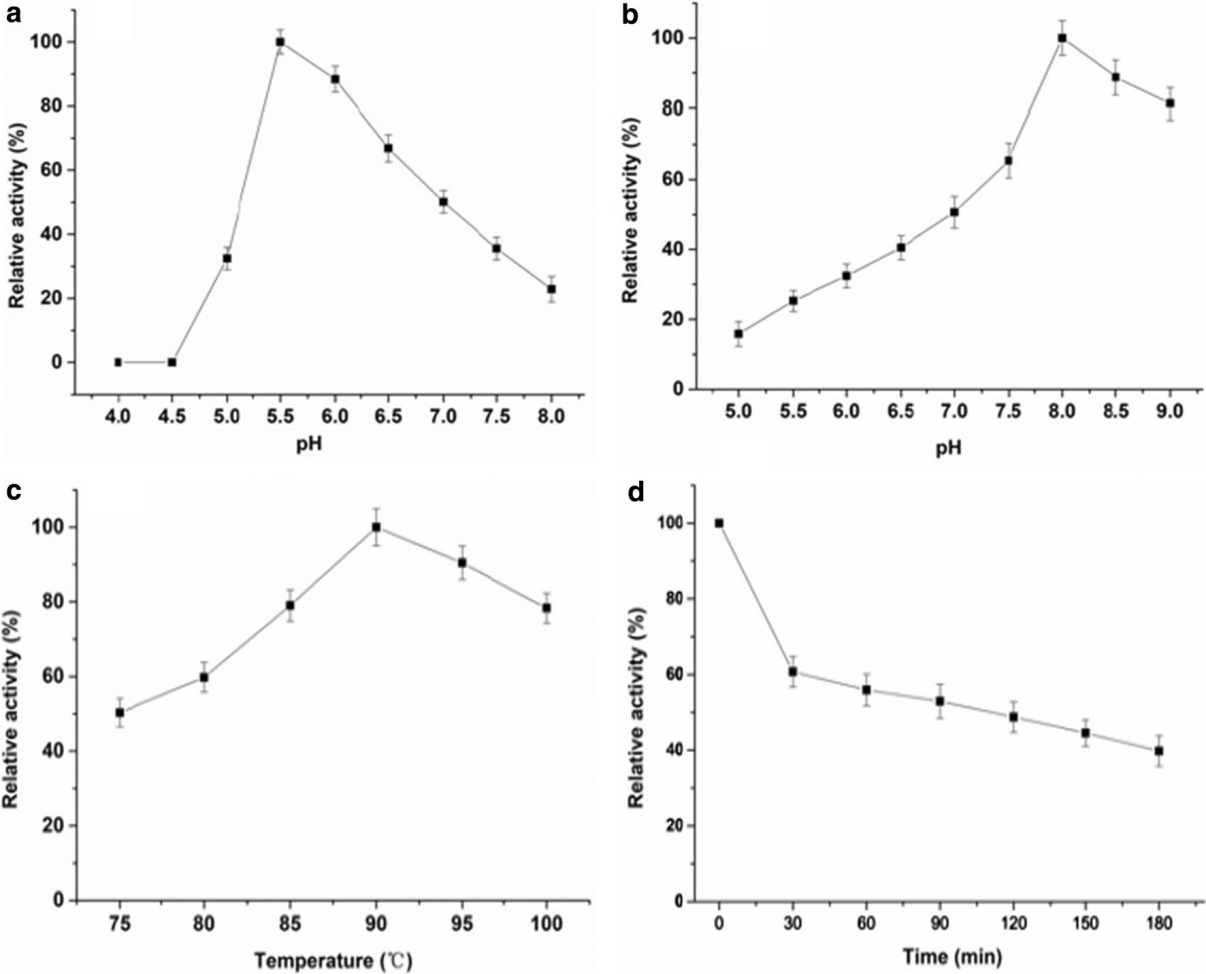
Effect of pH and temperature on TsCDase activity. **a**) Effect of pH on TsCDase activity. The maximum activity detected at pH 5.5 was taken as 100%. **b** Effect of pH on TsCDase stability. The maximum activity detected at pH 8.0 was taken as 100%. **c** Effect of temperature on TsCDase activity. The maximum activity detected at 90 °C was taken as 100%. **d** Thermostability of TsCDase. The thermostability of TsCDase was determined at 90 °C and TsCDase activity without incubation was taken as 100%

The effect of various pH (4.0–8.0) on TsCDase activity was investigated by using β-CD as the substrate. The optimal pH for enzyme activity was detected to be pH 5.5, and relative activity at the pH range 5.5–6.0 was over 60.0% of maximum activity (Fig. 3a). The pH effect on TsCDase stability was obtained by incubation at 4 °C and pH 5.0–9.0 for 24 h. Optimal TsCDase stability was observed at pH 8.0. Relative activity of TsCDase was stable at pH 7.5–9.0, retaining over 60.0% of maximum activity, suggesting TsCDase was suitable for storage in weak alkaline buffer. TsCDase was active in the range 75–100 °C with an optimal temperature of 90 °C (Fig. 3). The optimal temperature of TsCDase was significantly different from other CDases, which have optimal temperatures of 35–65 °C [7, 20, 21]. The thermostability of TsCDase was determined at 90 °C. TsCDase retained over 80.0% of maximum activity after incubation for 20 min, and its half-life was 120 min. These results revealed the optimal catalytic conditions of TsCDase were 90 °C and pH 5.5, which are the same as for amylopullulanases from thermophilic archaea [22].

### Substrate specificity of TsCDase

The specificity of TsCDase towards several substrates was given in Table 3. TsCDase showed its highest activity on β-CD in comparison to α-CD and γ-CD, and no activity on soluble starch and pullulan polysaccharide, suggesting the strong substrate specificity of TsCDase for CDs. Its substrate specificity was similar to that of CDases from *Paenibacillus* sp [23]. However, the CDases from *P. furiosus* [24] and *Thermofilum pendens* [25] were reported to hydrolyze not only CDs, but also pullulan and soluble starch, as they were identified as multispecific enzymes that possessed the activities of both α-amylase and a cyclodextrinase [26].

**Table 3:** Relative activity of TsCDase on different sustrates

Substrates	Relative activity (%)
β-CD	100
α-CD	80
γ-CD	73
Soluble starch	N.D.
Pullulan polysaccharide	N.D.

### Preparation of maltoheptaose by TsCDase

**Fig. 4:**
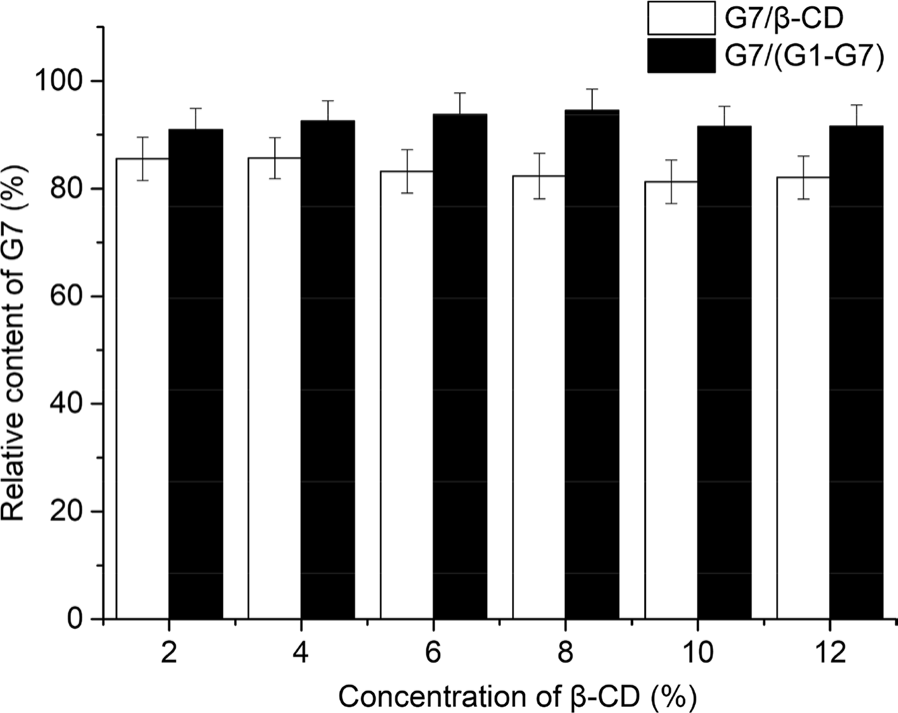
Influence of substrate concentration on preparation of maltoheptaose. Reaction condition was 90 °C, 150 rpm, pH 5.5 for 4 h with TsCDase (2.8 U) in final volume of 30 mL.  indicated yield of maltoheptaose in all reaction products.  indicated the proportion of maltoheptaose in malto-oligosaccharides

In this study, the preparation of maltoheptaose was investigated using different concentrations of β-CD as the substrate at 90 °C, pH 5.5 for 4 h. Insoluble β-CD was precipitated by adding acetonitrile to the reaction mixture at room temperature, which was consistent with a previous report [27]. As shown in Fig. 4, with increase of β-CD concentration, the production of maltoheptaose increased and achieved its maximum value at 8% β-CD (w/v) with an 82.33% for yield of maltoheptaose in all reaction products and 94.55% for the proportion of maltoheptaose in malto-oligosaccharides. Compared with the result described by Ji et al. [17], TsCDase exhibited greater product specificity, and converted β-CD to maltoheptaose at a high conversion rate (82.33%), which is the highest yield achieved in the preparation of maltoheptaose by CDases. Interestingly, when β-CD concentration was up to 12%, yield of maltoheptaose also retained over 80.0% by TsCDase, indicating TsCDase has significant product specificity and be active at high concentrations of β-CD with no substrate inhibition effect. Ji et al. reported that the enzymatic reaction of *P. pacificus* CDase was suppressed by a high concentration of β-CD [17], which is significantly difference from the results of this study.

## Conclusions

A cyclodextrinase (TsCDase) from *Thermococcus* sp B1001 was successfully expressed in *B. subtilis* for the first time. The results indicated that TsCDase had optimal activity at pH 5.5 and 90 °C, and was extremely thermostable. TsCDase has unique substrate and product specificities, which provide advantages in the preparation of maltoheptaose by using β-CD as substrate. When the concentration of β-CD was 8% under the the optimal conditions of 90 °C and pH 5.5 for 4 h, the maximum yield of maltoheptaose in all reaction products was 82.33%, and the proportion of maltoheptaose in the malto-oligosaccharides-products was 94.55%. Therefore, TsCDase has potential value in the preparation of maltoheptaose in the food industry as it can provide a specific polymerization degree of malto-oligosaccharides.

## Supplementary information

**Additional file 1:Figure S1.** Protein sequence analysis of cyclodextrins from various sources.

## Abbreviations

CDase: Cyclodextrinase
TsCDase: Cyclodextrinase from *Thermococcus* sp B1001
CDs: Cyclodextrins
α-CD: α-Cyclodextrin
β-CD: β-Cyclodextrin
γ-CD: γ-Cyclodextrin
*tscd*: Cyclodextrin hydrolase gene
DNS: 3,5-Dinitrosalicylic acid
SDS-PAGE: Sodium dodecyl sulfate–polyacrylamide gel electrophoresis

## References

[1] Marchal LM, Beeftink HH, Tramper J (1999). Towards a rational design of commercial maltodextrins. Ahan.

[2] Xu Q, Chao YL, Wan QB (2009). Health benefit application of functional oligosaccharides. Carbohydr Polym.

[3] Messaoud EB, Ali MB, Elleuch N, Masmoudi NF, Bejar S (2004). Purification and properties of a maltoheptaose- and maltohexaose-forming amylase produced by *Bacillus subtilis* US116. Enzyme Microb Technol.

[4] Kobayashi K, Sumitomo H, Itoigawa T (1987). Maltopentaose-and maltoheptaose-carrying styrene macromers and their homopolymers. Macromolecules.

[5] Li BG, Zhang LM (2008). Synthesis and characterization of novel amphiphilic block copolymers based on maltoheptaose and poly(ε-caprolactone). Carbohydr Polym.

[6] Park KH, Kim TJ, Cheong TK, Kim JW, Svensson B (2000). Structure, specificity and function of cyclomaltodextrinase, a multispecific enzyme of the α-amylase family. Biochim Biophys Acta.

[7] Sumio K, Michio T, Duque BS, Toshiyuki S, Shigetaka O (1983). Purification and some properties of cyclodextrinase from *Bacillus coagulans*. Agric Biol Chem.

[8] Turner P, Labes A, Fridjonsson OH, Hreggvidson GO, SchNheit P, Kristjansson JK, Holst O, Karlsson EN. Two novel cyclodextrin-degrading enzymes isolated from thermophilic bacteria have similar domain structures but differ in oligomeric state and activity profile. J Biosci Bioeng. 2005.100. 10.1263/jbb.100.380 16310726

[9] Nakagawa Y (2008). Gene cloning and enzymatic characteristics of a novel γ-cyclodextrin-specific. Biochim Biophys Acta Proteins Proteomics.

[10] Tachibana Y, Kuramura A, Shirasaka N, Suzuki Y, Yamamoto T, Fujiwara S, Takagi M, Imanaka T (1999). Purification and characterization of an extremely thermostable cyclomaltodextrin glucanotransferase from a newly isolated hyperthermophilic archaeon, a *Thermococcus* sp. Appl Environ Microbiol.

[11] Hashimoto Y, Yamamoto T, Fujiwara S, Takagi M, Imanaka T (2001). Extracellular synthesis, specific recognition, and intracellular degradation of cyclomaltodextrins by the hyperthermophilic archaeon *Thermococcus* sp. strain B1001. J Bacteriol.

[12] You C, Zhang XZ, Zhang YH (2012). Simple cloning via direct transformation of PCR product (DNA Multimer) to *Escherichia coli* and *Bacillus subtilis*. Appl Environ Microbiol.

[13] Turgeon N, Laflamme C, Ho J, Duchaine C (2006). Elaboration of an electroporation protocol for *Bacillus cereus* ATCC 14579. J Microbiol Methods.

[14] Laemmli UK (1970). Cleavage of structural proteins during the assembly of the head of bacteriophage T4. Nature.

[15] Bradford MM (1976). A rapid and sensitive method for the quantitation of microgram quantities of protein utilizing the principle of protein-dye binding. Anal Biochem.

[16] Bernfeld P (1955). Amylases alpha and beta. Methods Enzymol.

[17] Ji H, Bai Y, Li X, Wang J, Xu X, Jin Z (2019). Preparation of malto-oligosaccharides with specific degree of polymerization by a novel cyclodextrinase from *Palaeococcus pacificus*. Carbohydr Polym.

[18] Lee MH, Yang SJ, Kim JW, Lee HS, Kim JW, Park KH (2007). Characterization of a thermostable cyclodextrin glucanotransferase from *Pyrococcus furiosus* DSM3638. Extremophiles.

[19] Li X, Li D (2015). Preparation of linear maltodextrins using a hyperthermophilic amylopullulanase with cyclodextrin- and starch-hydrolysing activities. Carbohydr Polym.

[20] Podkovyrov SM, Zeikus JG (1992). Structure of the gene encoding cyclomaltodextrinase from *Clostridium thermohydrosulfuricum* 39E and characterization of the enzyme purified from *Escherichia coli*. J Bacteriol.

[21] Kim TJ, Shin JH, Oh JH, Kim MJ, Lee SB, Ryu S, Kwon K, Kim JW, Choi EH, Robyt JF (1998). Analysis of the gene encoding cyclomaltodextrinase from Alkalophilic *Bacillus* sp. I-5 and characterization of enzymatic properties. Arch Biochem Biophys.

[22] Li X, Li D, Park KH (2013). An extremely thermostable amylopullulanase from *Staphylothermus marinus* displays both pullulan- and cyclodextrin-degrading activities. Appl Microbiol Biotechnol.

[23] Kaulpiboon J, Pongsawasdi P (2004). Expression of cyclodextrinase gene from *Paenibacillus* sp. A11 in *Escherichia coli* and characterization of the purified cyclodextrinase. J Biochem Mol Biol.

[24] Yang SJ, Lee HS, Park CS, Kim YR, Moon TW, Park KH (2004). Enzymatic analysis of an amylolytic enzyme from the hyperthermophilic archaeon *Pyrococcus furiosus* reveals its novel catalytic properties as both an alpha-amylase and a cyclodextrin-hydrolyzing enzyme. Appl Environ Microbiol.

[25] Li X, Li D, Yin Y, Park KH (2010). Characterization of a recombinant amylolytic enzyme of hyperthermophilic archaeon *Thermofilum pendens* with extremely thermostable maltogenic amylase activity. Appl Microbiol Biotechnol.

[26] Koo YS, Ko DS, Jeong DW, Shim JH (2017). Development and application of cyclodextrin hydrolyzing mutant enzyme which hydrolyzes β- and γ-CD selectively. J Agric Food Chem.

[27] Grard S, Elfakir C, Dreux M (2000). Characterization of sulfobutyl ether-beta-cyclodextrins mixtures by anion-exchange chromatography using evaporative light scattering detection. J Chromatogr A.

