# Supplementary material for: Cyclodextrinase from Thermococcus sp expressed in Bacillus subtilis and its application in the preparation of maltoheptaose
Source: Microb Cell Fact. 2020 Aug 1;19:157. doi: 10.1186/s12934-020-01416-y (PMC7395394; doi:10.1186/s12934-020-01416-y)

Cyclodextrinase from *Thermococcus* sp expressed in *Bacillus subtilis* and its application in the preparation of maltoheptaose

Figure S1 Protein sequence analysis of cyclodextrins from various sources

CDase: Cyclomaltodextrinase; NPase: Neopullulanase; MAase: Maltogenic amylase; Square brackets: Gene accession number

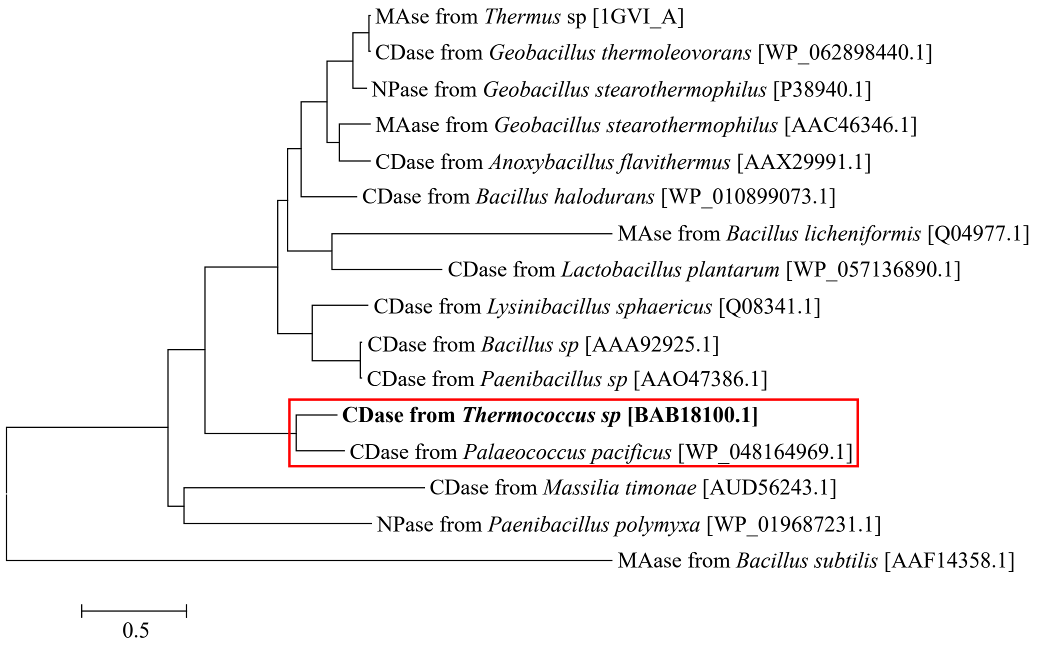

Supplement: Supplementary file 1 — Additional file 1:Figure S1. Protein sequence analysis of cyclodextrins from various sources. [file 12934_2020_1416_MOESM1_ESM.pdf]
